# Development of novel SSR markers for evaluation of genetic diversity and population structure in *Tribulus terrestris* L. (Zygophyllaceae)

**DOI:** 10.1007/s13205-016-0469-8

**Published:** 2016-07-19

**Authors:** Kuljit Kaur, Vikas Sharma, Vijay Singh, Mohammad Saleem Wani, Raghbir Chand Gupta

**Affiliations:** Department of Botany, Punjabi University, Patiala, Punjab 147002 India

**Keywords:** *Tribulus terrestris*, Simple Sequence Repeats (SSRs), Polymorphism, Genetic diversity, Population structure

## Abstract

*Tribulus terrestris* L., commonly called puncture vine and gokhru, is an important member of Zygophyllaceae. The species is highly important in context to therapeutic uses and provides important active principles responsible for treatment of various diseases and also used as tonic. It is widely distributed in tropical regions of India and the world. However, status of its genetic diversity remained concealed due to lack of research work in this species. In present study, genetic diversity and structure of different populations of *T. terrestris* from north India was examined at molecular level using newly developed Simple Sequence Repeat (SSR) markers. In total, 20 primers produced 48 alleles in a size range of 100–500 bp with maximum (4) fragments amplified by TTMS-1, TTMS-25 and TTMS-33. Mean Polymorphism Information Content (PIC) and Marker Index (MI) were 0.368 and 1.01, respectively. Dendrogram showed three groups, one of which was purely containing accessions from Rajasthan while other two groups corresponded to Punjab and Haryana regions with intermixing of few other accessions. Analysis of molecular variance partitioned 76 % genetic variance within populations and 24 % among populations. Bayesian model based STRUCTURE analysis detected two genetic stocks for analyzed germplasm and also detected some admixed individuals. Different geographical populations of this species showed high level of genetic diversity. Results of present study can be useful in identifying diverse accessions and management of this plant resource. Moreover, the novel SSR markers developed can be utilized for various genetic analyses in this species in future.

**Electronic supplementary material:**

The online version of this article (doi:10.1007/s13205-016-0469-8) contains supplementary material, which is available to authorized users.

## Introduction

**Fig. 1:**
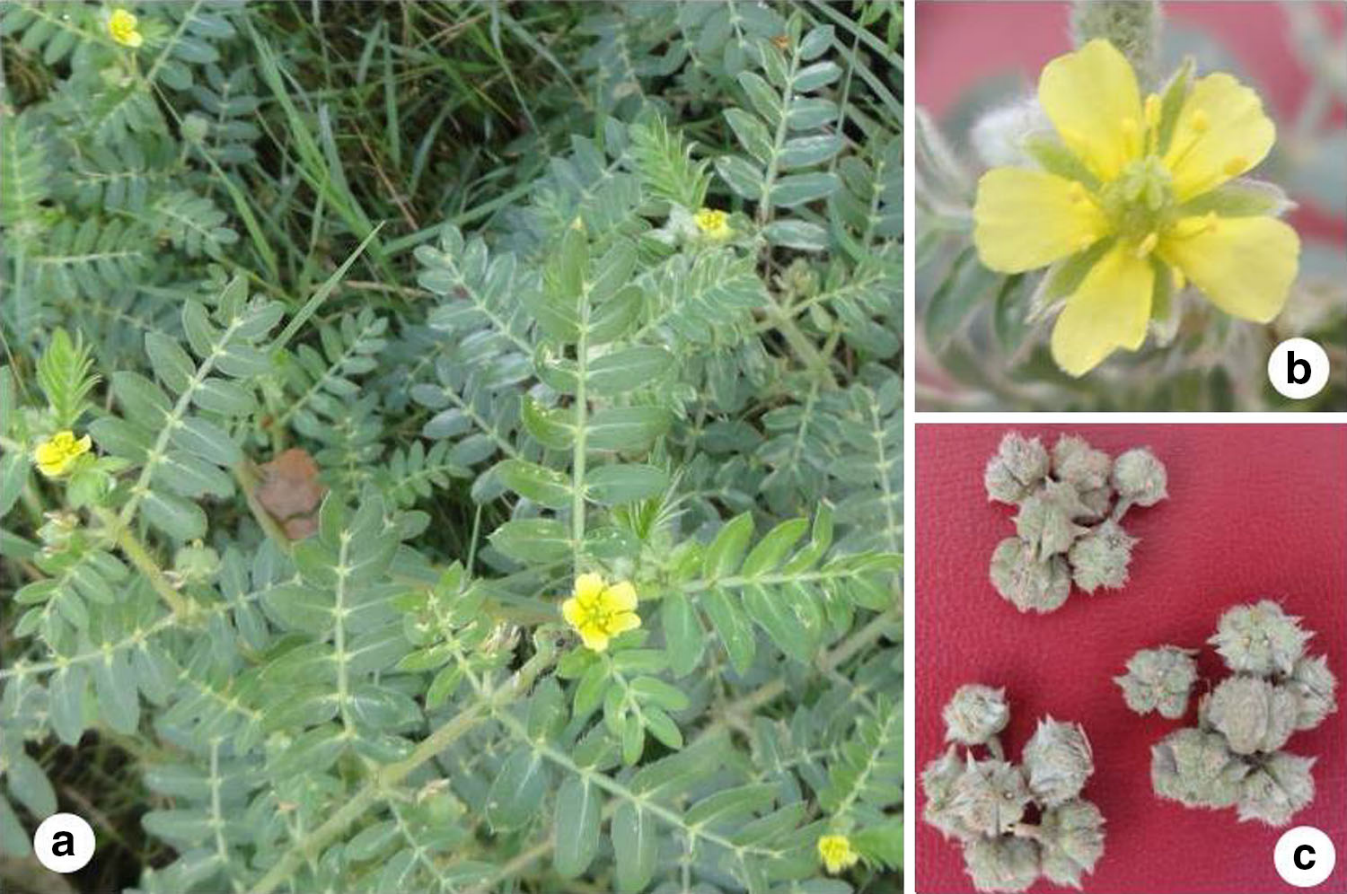
**a** A photo of plant at natural habitat of Punjab showing plant bearing leaves and flowers. **b** Enlarged photo of flower. **c** Seeds of *T. terrestris*

*Tribulus terrestris* commonly known as puncture vine and Gokhru, belonging to family Zygophyllaceae, is an annual herbaceous plant. The plant is native to the South and East Europe and West Asia. It is widely distributed in the warm regions of Asia, Africa, Europe, America and Australia (Topia et al. 1994; Abeywickrama and Bean 1991; Kostova et al. 2002). The species is commonly distributed throughout tropical and warmer regions of India. It occurs naturally in many Indian states with warm climate and reported from eastern, western, northern, southern and central parts of country (Mishra and Bisht 2012; Das and Ghosh 2014; Fatima et al. 2014; Pandey 2014, 2015). It is a prostrate to procumbent annual, hairy herb. Leaves are pinnately compound, leaflets are 4–8 paired, subsessile, ovate or elliptic. Flowers are yellowish colored, mericarps not winged but distinctly spinous (Fig. 1). The fruits of the species are very distinguished in nature like a stellate and are known as ‘Chih-hsing’ in China and ‘Goat head’ in USA. It is used as traditional ayurvedic medicine in various health disorders. It contains saponins, steroids, estradiol, flavonoids, alkaloids, unsaturated fatty acids, vitamins, tannins, resins, nitrate potassium, aspartic acid and glutamic acid (Gauthaman and Adaikan 2005). The plant is a rich source of saponins, of which protodioscin has received a large attention in regards to sexual dysfunction issues (Adimoelja 2000). Now-a-days, full plant or its fruits are used in large number of purposes as skin-care, human hormone regulation, antibacterial, anti-inflammation, antivirus and immunostimulant. The whole plant is useful in strangury, dyspepsia, helminthiasis, cough, asthma, cardiopathy, skin diseases, hypertension and rheumatic arthritis (Sivarajan and Balachandran 1994; Warrier et al. 1996; Petkov 2011). Thus, it is well known that all parts of the plant have great medicinal potential. *T. terrestris* exhibits both, self- and cross-pollination mechanisms (Ganie 2011). This herb propagates through the seeds only. The species is dibasic depending on base number *x* = 6 and 10. Cytologically, the species is quite variable with intra-specific polyploids from diploid to octaploid (2*n* = 12, 24, 36, 48; Morrison and Scott 1996). From India, the chromosome count of 2*n* = 24, 32, 36, 48, 72, 96 has been reported by various workers. From Rajasthan, three cytotypes of the species, i.e., tetraploid, hexaploid and octaploid have been reported (Gupta et al. 2016). Morrison and Scott (1996) suggested that these various polpyploids are originated from allopolyploid complex. Propagation through seeds presents opportunity for analyzing polymorphisms of these populations which are not so far apart, as reproductive mode results in allelic recombinations. In northern parts of India, this species is widely found in Punjab, Haryana and Rajasthan. As it requires very less water for its growth, it is commonly found in bare and uncultivated lands. Although, *T. terrestris* is cultivated in various regions of Rajasthan for its medicinal utilities but these practices further needs elite germplasm for sustainable utilization. On the other, due to preference to major cereal crops and other cash crops in Punjab and Haryana, populations of this species often uprooted in large scale which is posing a threat to genetic diversity of this species. Therefore, it becomes imperative to characterize its existing germplasm so that diverse germplasm can be identified and maintained for future.

To study genetic diversity and population structure at molecular level different types of DNA markers can be used. However, Simple Sequence Repeats (SSRs; Tautz 1989) and Single Nucleotide Polymorphism (SNP) markers are the most preferred markers now-a-days. These DNA markers are frequently applied for genetic diversity evaluation, population structure assessment, for inferring interrelationships and linkage mapping. SSR markers are multiallelic, evenly distributed in genome, cross-transferable in related species and genera, and are amenable to automation. SNP markers are mostly biallelic, had higher frequency in genome and exhibit high heritability (Gaur et al. 2012; Verma et al. 2015). SNP markers are less polymorphic than SSRs but their abundance in genome overcomes this limitation (Mammadov et al. 2012). As both these markers represent different genomic regions they can provide different views for a similar investigation (Singh et al. 2014). SNP markers require prior sequence information while the cross-transferable nature of SSR markers makes them more favorable and useful for genetic analysis in related species lacking sequence data. Furthermore, Mesak et al. (2014) by comparing these two markers for characterization of clonal lineages in fish suggested that SNP data sets demand more caution and stringency to achieve good results and to reduce false signals. Thus, SSR appears more favourable and accessible to conduct genetic analysis in non-modal plant species, as SNP requires sophisticated instrumentation for their validation. Therefore, in present study we utilized *T. terrestris* sequence data available at National Centre for Biotechnology Information (NCBI) for designing new SSR markers to evaluate the genetic diversity and population structure of this species in north India.

## Materials and methods

### Plant material and DNA extraction

In present study twenty-six *T. terrestris* accessions collected from different geographical locations of three north Indian states (Punjab, Haryana and Rajasthan) were analyzed. Of these, twelve samples were from Punjab, six from Haryana and eight from Rajasthan. A detatiled account of all the accessions is given in Table 1. Young fresh leaves were collected from each sample for isolation of DNA. DNA was extracted following the CTAB method (Doyle and Doyle 1990). DNA stocks preparation in Tris (10 mM) EDTA (1 mM) buffer, quantification and dilutions for making working stocks was done according to Sharma et al. (2015).

**Table 1:** List of *Tribulus terrestris* genotypes used in present study. Their location with elevation is also shown

S. no.	Genotype	Location	Ploidy level	Altitude (m)
1	Raj-01	Pali	Hexploid	230
2	Raj-02	Hemawas Dam, Pali	Tetraploid	237
3	Raj-03	BSI, Jodhpur	Octaploid	282
4	Raj-04	Sikar	–	420
5	Raj-05	Gajsar, Churu	–	292
6	Raj-06	Sri Ganganagar	–	164
7	Raj-07	Neemrana, Alwar	Tetraploid	332
8	Raj-08	Rai Singhnagar	–	165
9	Pun-01	Urban Estate, Patiala	–	256
10	Pun-02	Bahadurgarh	–	260
11	Pun-03	Professor colony, Patiala	–	250
12	Pun-04	Chotti baradari, Patiala	–	250
13	Pun-05	Chandigarh	–	350
14	Pun-06	Zirakpur	–	350
15	Pun-07	Punjabi University, Patiala	Tetraploid	250
16	Pun-08	RS Patiala	–	250
17	Pun-09	Rajpura	–	259
18	Pun-10	Patiala	–	250
19	Pun-11	43 Chandigarh	–	350
20	Pun-12	Mohali	–	315
21	Har-01	Panchkula	–	365
22	Har-02	Panchkula	Octaploid	365
23	Har-03	Panchkula	–	365
24	Har-04	Ambala	–	270
25	Har-05	Ambala Cantt	–	264
26	Har-06	Ambala	–	270

### Sequence data and primer designing

Sequence data at NCBI was searched for *T. terrestris*. A total of 637 nucleotide sequences were available as on 12th May 2015. These nucleotide sequences were retrieved and assembled using EGassembler (Masoudi-Nejad et al. 2006) to make final consensus sequences and to remove the redundancy. The resulted contigs and singletons were then searched for presence of SSRs with minimum repeat length of 10 bp using SSRIT (Temnykh et al. 2001). SSR primers were designed from suitable SSR containing sequences using PRIMER 3 software (Rozen and Skaletsky 2000) with default settings. These SSRs were named as- *Tribulus terrestris* MicroSatellite (TTMS).

### SSR genotyping

PCR reactions were performed in a 10 µL reaction volume as per Sharma et al. (2009). PCR amplification was carried out in Biorad Thermal Cycler (Biorad, Australia) and all PCR reactions were performed at one cycle of 4 min at 94 °C as initial denaturation, followed by 35 cycles with a denaturation step at 94 °C for 30 s, an annealing step for 45 s at respective annealing temperature of each primer in a range of 51–55 °C (Table 2), and an extension step at 72 °C for 1 min, followed by last cycle of extension at 72 °C for 7 min. PCR products were resolved on 3 % agarose gel and size of each fragment was estimated using 50 bp ladder (MBI Fermentas, Lithuania). Gels were prepared and run in 1X TBE buffer and visualization of fragments was done using ethidium bromide. Permanent photographs of gels were taken in gel documentation system (Bio-Rad laboratories-segrate, Milan, Italy).

**Table 2:** Newly designed and characterized SSR primers with their diversity characteristics

Primer name	Primer sequence (5′–3′)	Repeat motif	*T*_*a*_ (°C)	No. of bands	Size range (bp)	PIC	MI
TTMS-1	F: TCCTCCAGGATGACAAGGTC R: AGTTCAGTGGTTGGGAGGTG	(TCT)4	55	4	200–500	0.453	1.81
TTMS-2	F: CAATCATCAATCCTCGCTGA R: TAGCTTCAGATGGGGTCTGG	(TTGT)3	51	3	150–400	0.405	1.21
TTMS-3	F: ATGCTGCTTCTCCCAGCTAA R: TCCTTCTAAGGCACCCACAC	(GCA)4	51	2	200–400	0.488	0.98
TTMS-4	F: CGTTGCTGCCCATCATATC R: CCGTTTATCAATGGCTCCAA	(TGC)4	51	3	250–450	0.381	1.14
TTMS-5	F: CCCACTCCCACTACCATCG R: CCATGATGATTTGGCACTGA	(CCACTT)4	55	2	100–200	0.453	0.91
TTMS-7	F: GGAATCGATGGCACGTAAAC R: TTCGACTGGGCAAGTCTTCT	(TTG)5	51	2	250–300	0.499	1.00
TTMS-8	F: GGAAATTCTCGGTGGTCTGA R: TGACAAGAGCATTGAAGTCGTC	(GTT)5	53	2	250–350	0.142	0.28
TTMS-10	F: CAGAGCGTTGAGAGGTTTTG R: CCATCCTTGGCAGTGAAAGT	(AGAGA)3	53	3	100–450	0.224	0.67
TTMS-11	F: GCTCGTAAAGGCCAAGACTG R: TAAAACCCCCATGCTCAATC	(AAAG)3	53	2	350–450	0.286	0.57
TTMS-12	F: GATTGAAGTTCCAGGCCAAG R: TCGTCTTTGTGGAAGCAAAT	(TTA)5	51	3	150–300	0.479	1.44
TTMS-13	F: CCTTGCCCCATCAAAGTTTA R: GTCTAGCAGCGGAAGGTCAT	(TTTAT)3	53	3	150–450	0.355	1.07
TTMS-19	F: CATTGTCTCCCCTCCAACAC R: GGCCAATCTCGAGCAGAATA	(CTAA)3	53	1	130	–	–
TTMS-22	F: AAGGGGAGTTGGTGGATTTC R: TACAGGACCAGCACTGCAAG	(TGTTG)3	51	2	250–350	0.355	0.71
TTMS-24	F: CCCCAAATAAGGCAAAAACA R: CGGCTGCAGCTATTAGAGGA	(CAA)5	51	2	250–350	0.174	0.35
TTMS-25	F: GAACAAGTTGTAAAGCACAGACC R: GATGACGAGAATGCCCTTGT	(GAA)6	53	4	100–490	0.488	1.95
TTMS-28	F: GACTTCTTCGCAGACTTTTCG R: TTCTCGAATACCTCGCTGCT	(AGC)5	53	1	120	–	–
TTMS-30	F: GCACCAAGAAACAAACAACG R: ACTGATGAGCTGCTCGAGGT	(TCT)4	51	2	100–300	0.393	0.79
TTMS-31	F: AACGACCGTTGTTGACATGA R: CGCTTGTGGTAGAGGGAGAG	(GTT)4	53	1	300	–	–
TTMS-32	F: AGACGCTACCGGACAACACT R: CAGCAAATGGCTTCTTCCAT	(GCG)5	51	2	150–250	0.233	0.47
TTMS-33	F: GCAACAAACTCTTCCGCTTC R: CAGCTTTTCCGCTCTCAAGT	(GAT)5	53	4	150–450	0.458	1.83
Mean				2.4		0.368	1.01

*T*_*a*_ Annealing temperature, *bp* base pair, *PIC* Polymorphism Information Content, *MI* Marker index

### Data analysis

All SSR fragments were scored manually and converted into binary data, i.e., 1 for presence of band and 0 for absence of band. Polymorphism information content (PIC) was calculated using formula given by Roldán-Ruiz et al. (2000), i.e., PICi = 2fi (1 − fi). Where PICi is the polymorphic information content of marker i, fi is the frequency of the marker bands present and (1 − fi) is frequency of marker bands absent. Marker index (MI) was calculated by applying the formula given by Prevost and Wilkinson (1999). Distance-based cluster analysis was performed and dendrogram based on the unweighted pair group method of arithmetic mean (UPGMA) was constructed using Jaccard’s similarity coefficient with the help of DARwin (Perrier and Jacquemoud-Collet 2006). Bayesian clustering methods are powerful computational tools meant for estimation of various features of population. STRUCTURE, which is a Bayesian clustering software assigns the individuals to different populations and hybrid zones on the basis of allele frequencies of genotypes. The method assumes K (unknown) populations for the given data set and the value of K can be estimated by posterior probability of the data for a given K, Pr (*X*|*K*) as per Pritchard et al. (2000). STRUCTURE software, version: 2.3.3 (Pritchard et al. 2000; Falush et al. 2007) was used to assess the genetic structure at population level as well as to detect genetic stocks contributing to this germplasm collection. Ancestry model with admixture and correlated allele frequency model was set to get the estimates of posterior probability of data. Ten independent runs were given setting the value of K from 1 to 5 with three iterations for each value of K. Both, length of burn-in period and number of Markov Chain Monte Carlo (MCMC) repeat after burn-in was set at 100,000. Evanno’s method (Evanno et al. 2005) based program STRUCTURE HARVESTER developed by Earl and Vonholdt (2011) was used to determine the value of estimated Ln probability of data-LnP(*K*) and to get the best fit value of *K* for the data. Highest value was shown at *K* = 2. Therefore, STRUCTURE analysis was conducted for *K* = 2. However, we also performed STRUCTURE analysis at *K* = 3 to compare the different clusters in relation to the groupings of dendrogram. Genetic differentiation (*F*_st_) estimates among inferred clusters were also measured by STRUCTURE software. Genetic relationships among the genotypes were also analyzed by factorial analysis using the software DARwin (Perrier and Jacquemoud-Collet 2006). Analysis of molecular variance (AMOVA) was performed with the help of GenAlex version 6.41 (Peakall and Smouse 2006).

## Results

### SSR polymorphism

Only unambiguous and reliable fragments amplified by 20 SSR primers were scored. In total, 20 primers amplified 48 fragments ranging from 1 to 4 with an average of 2.4 fragments. Size range of amplified fragments varied from 100 to 500 bp. Three primer namely, TTMS-19, TTMS-28 and TTMS-31 amplified minimum of 1 fragment while three primers namely, TTMS-1, TTMS-25 and TTMS-33 amplified maximum of 4 fragments. PIC value ranging from 0.142 for primer TTMS-8 to 0.499 for primer TTMS-7 with an average of 0.368 (Table 2). Marker index value was highest (1.95) in TTMS-25 and lowest (0.28) in TTMS-8 with a mean of 1.01 as shown in Table 2.

### Cluster and diversity analysis

**Fig. 2:**
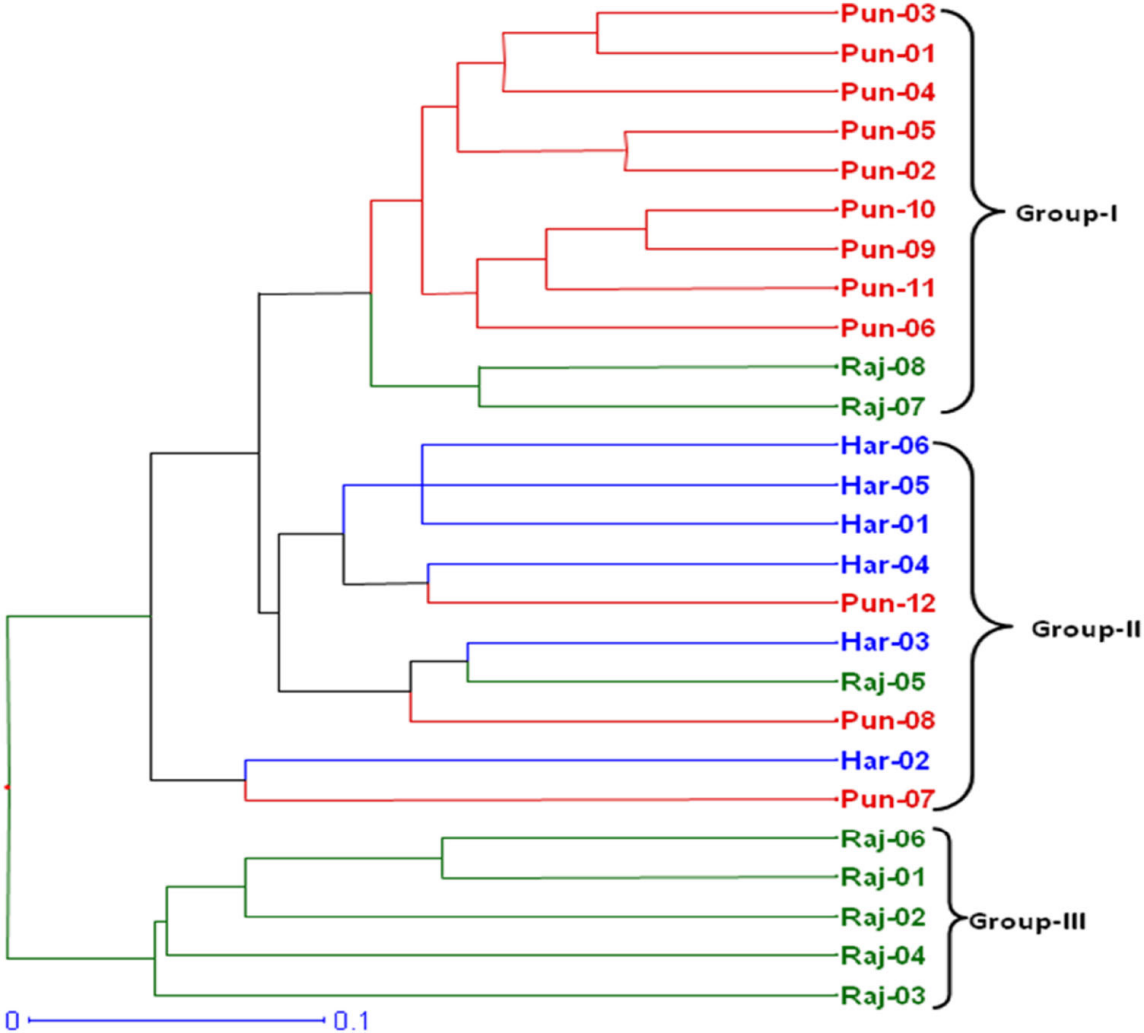
Dendrogram of 26 *T. terrestris* accessions showing clustering of all accessions into three major groups respective to their geographical locations

**Fig. 3:**
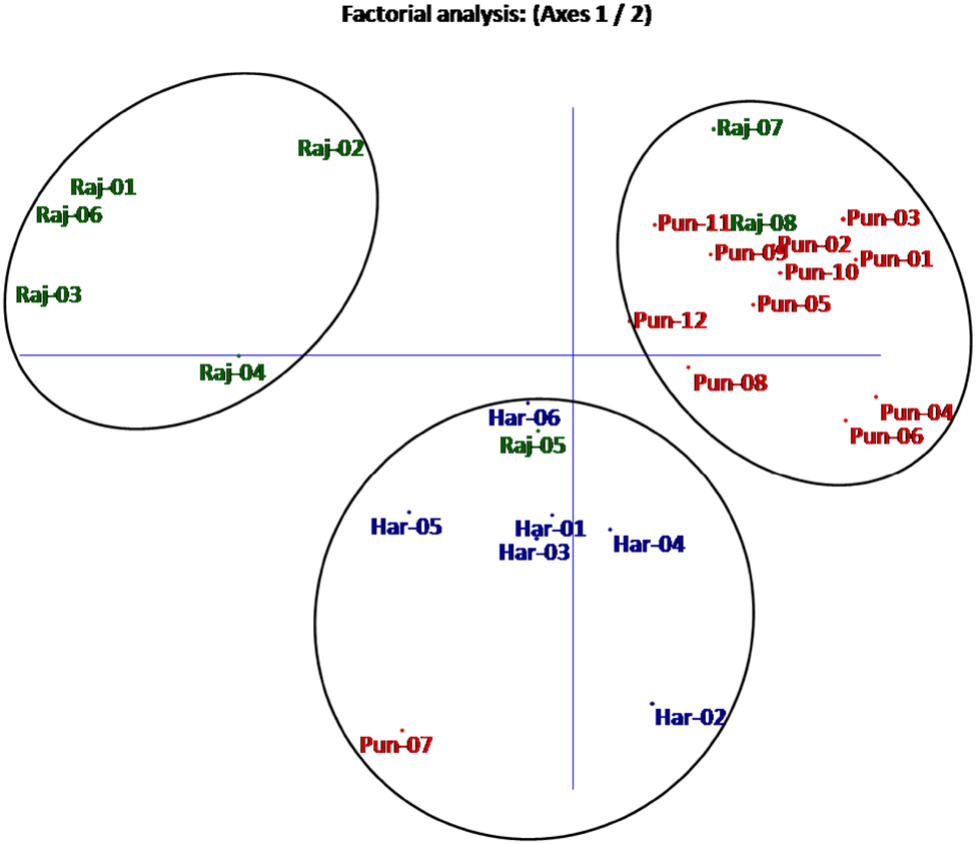
Factorial analysis based on eigen values calculated from 20 SSR markers. The 26 accessions were clustered into three geographical populations as shown by *different colors*

Dendrogram based on Jaccards similarity coefficient and UPGMA method showed three groups as shown in Fig. 2. Group-I included 11 accessions and represented majority (9) of accessions from Punjab and exceptionally included two accessions (Raj-07 and Raj-08) from Rajasthan. Group-II included 10 mixed accessions from all the three states but majority (6) of accessions was from Haryana state. The third smallest group-III contained all the five accessions from Rajasthan forming a pure group. The maximum genetic similarity value was 0.87 between Pun-09 from Rajpura and Pun-10 from Patiala showing them the most similar accessions whereas Raj-03 from Jodhpur and Pun-04 from Chhoti Baradari were found to be genetically most dissimilar with the minimum similarity value of 0.270. Dendrogram largely distinguished the different accession on the basis of their locations with few exceptions. Two dimensional graphical view of genetic diversity in 26 analyzed accessions was represented in factorial analysis (Fig. 3) which showed clearly the three groups of *T. terrestris* accessions. Clustering patterns in factorial supported clustering of both dendrogram and STRUCTURE. Further, AMOVA analysis revealed that the major (76 %) portion of genetic variation resided within different populations while 24 % genetic variance resided among populations.

### Bayesian genetic structure

**Fig. 4:**
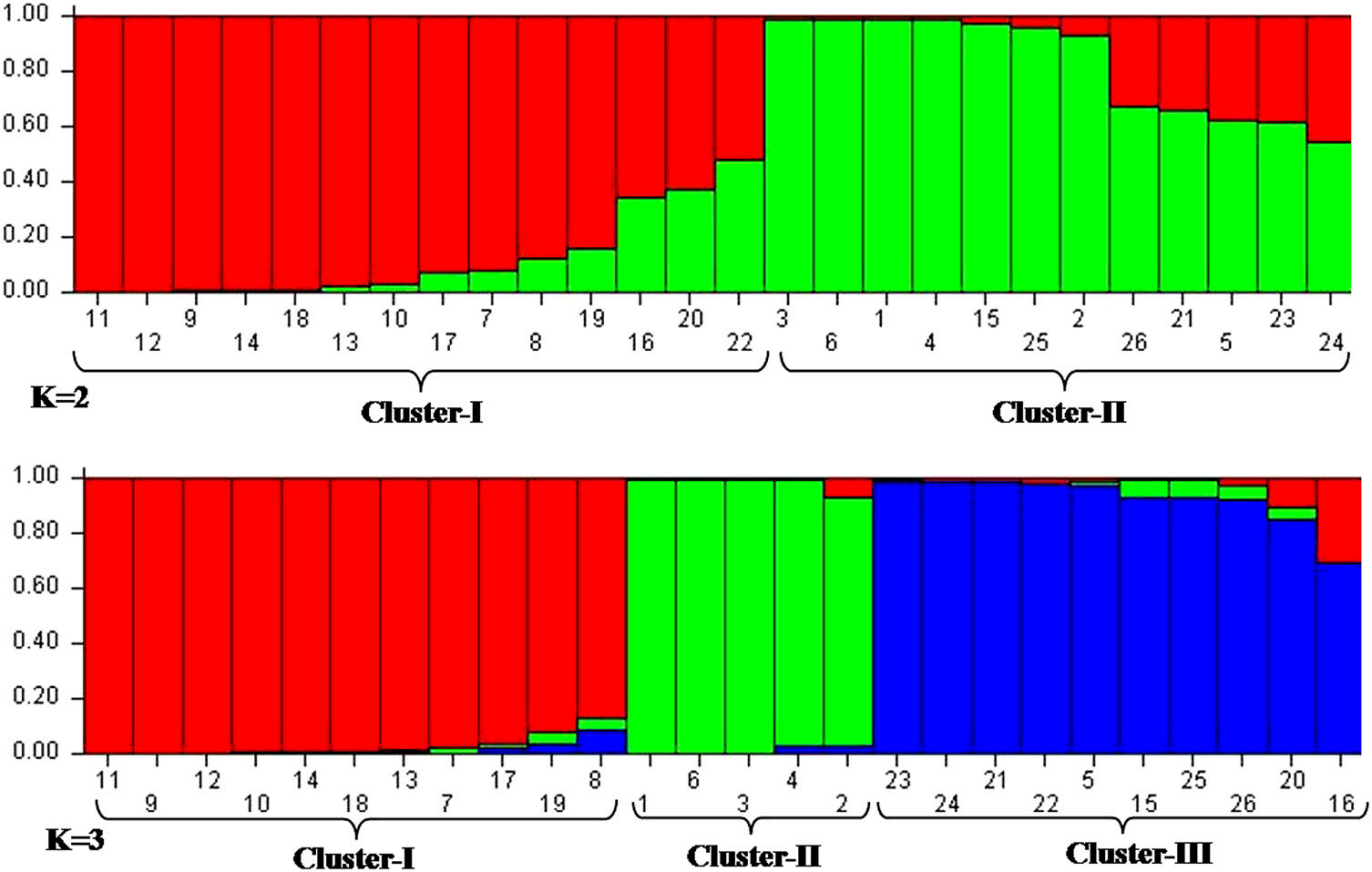
Twenty six accessions of *T. terrestris* assigned into different clusters by STRUCTURE. **a** At *K* = 2. **b** At *K* = 3

The structure harvester computed best value of *K* at 2. However, when we analyzed data at *K* = 3, the clustering largely supported the grouping of dendrogram. At *K* = 2, all the accessions, namely, Pun-01, Pun-02, Pun-03, Pun-04, Pun-05, Pun-06, Pun-08, Pun-09, Pun-10, Pun-11, Pun-12, belonging to Punjab clustered in cluster-I except one accession namely, Pun-07 which was collected from Punjabi University campus. On the other hand, the maximum accessions belonging to Rajasthan (Raj-01, Raj-02, Raj-03, Raj-04, Raj-05 and Raj-06) and Haryana (Har-01, Har-03, Har-04, Har-05 and Har-06) clustered in cluster-II. Mean values of *F*_st_ for cluster-I and cluster-II were 0.443 and 0.440, respectively. Percentages of pure accessions in cluster-I and cluster-II were 64.2 and 58.3 %, respectively (Fig. 4). However, at *K* = 3, some of the accessions from the Rajasthan clustered separately and formed a pure cluster representing *T. terrestris* germplasm from Rajasthan.

## Discussion

### SSR diversity

DNA markers offer more accurate information on genetic diversity, as they are rarely influenced by environmental factors of a region (Sharma et al. 2008). Furthermore, SSR markers specifically possess many desirable useful properties which makes them most preffered DNA markers (Powell et al. 1996; Rana et al. 2015; Sharma et al. 2015). The newly developed SSR markers appeared valuable in detecting diversity in studied germplasm of *T. terrestris*. Twenty SSR primers generated 2.4 fragments on an average. The less numbers of alleles detected in this study may be attributed to the analyzed populations which were not so apart geographically from each other. In addition, the designed SSRs were from very less sequence data which can be from conserved and stable regions of DNA with regards to mutation rates. Eight SSR primers amplified at least three or more than three fragments and eight primers showed a PIC value greater than 0.4 which is an indicator of high polymorphism. The high genetic diversity and polymorphism observed in present study is in concordance to results of Sarwat et al. (2008). Average PIC value of 0.368 was found higher than obtained in different populations of *Valeriana jatamansi* and *Salvadora oleoides* by Jugran et al. (2015) and Yadav et al. (2014), respectively. However, this mean PIC value was comparable to the values detected in other medicinal plants by various workers (Shaw et al. 2009; Rostami-Ahmadvandi et al. 2013; Tabin et al. 2016). The average number of fragments detected per primer and, PIC and MI values showed that newly developed and characterized marker proved useful in revealing polymorphism in different populations of *T. terrestris*. The maximum genetic similarity value of 0.870 was observed between Pun-09 from Rajpura and Pun-10 from Patiala showing them the most similar accessions. As these two regions are not very far, high similarity is obvious. It further, indicate that even having cross-pollination and seed dispersal mechanisms, somehow this species is maintaining homozygosity in its populations which requires further investigations. The most diverse accessions were Raj-03 from Jodhpur and Pun-04 from Chhoti Baradari with the minimum similarity value of 0.270. The genetic distantness of these two accessions indicates high diversity in different germplasm prevailing in studied areas.

### Cluster analysis and genetic structure

Various clustering methods give the estimate of genetic relatedness of analyzed accessions of a species. Among different clustering tools, the tools based on Baysean methods are considered more robust. Here we used both distance-based and simulation-based Baysean methods for inferring genetic relationships and structure of different *T. terrestris* populations. Distance-based dendrogram constructed using Jaccards similarity coefficient and UPGMA method grouped all accessions into three groups. Broadly these three groups corresponded to geographical locations of the accessions but group-I which represented majority of accessions from Punjab and group-II which represented majority of accessions from Haryana state, exceptionally included few accessions from other regions. This showed that besides, geographic isolation, different populations of this species are interrelated by some means or mechanisms which is beneficial for enhancing its genetic diversity. One small group (group-III) purely represented accessions from Rajasthan indicating conserved genetic nature of dry region germplasm. Furthermore, as this germplasm is cultivated repeated propagation of same parent germplasm may have lead to its conservative and more homozygous nature. Few exceptional groupings needs further research and analysis. Factorial analysis (Fig. 3) supported the groupings by dendrogram and showed three groups of *T. terrestris* accessions in which cluster representing Punjab region was having more intermixing of accessions. AMOVA analysis in present study revealed that the major portion of genetic variation resided within different populations of *T. terrestris* (76 %) rather than among populations (24 %). It points that allele sharing is more frequent within populations than in between populations. Baysean structure analysis indicated two genetic stocks for the entire germplasm included in present study. Cluster-I (Pun-01, Pun-02, Pun-03, Pun-04, Pun-05, Pun-06, Pun-08, Pun-09, Pun-10, Pun-11, Pun-12) belonging to Punjab showed that genetic background of accessions from Punjab was different than the accessions (Raj-01, Raj-02, Raj-03, Raj-04, Raj-05, Raj-06, Har-01, Har-03, Har-04, Har-05 and Har-06) from other two states which clustered in cluster-II. Mean values of Fst for cluster-I and cluster-II were 0.443 and 0.440, respectively, which were much higher than reported by Duran et al. (2005) in Creosotebush (Zygophyllaceae: *Larrea tridentata*) populations and by Fuchs and Hamrick (2010) in *Guaiacum sanctum* (Zygophyllaceae). This showed that genetic differentiation was high in analyzed accessions of *T. terrestris* than reported in the other members of family. Relative high percentages of pure accessions in both clusters indicate low admixture and natural interbreeding of accession. The reason may be distantness of the accessions included in the study. At *K* = 3, some of the accessions from the Rajasthan clustered separately and formed a pure cluster representing *T. terrestris* germplasm from Rajasthan. These observations showed that although there present only two genetic stocks for entire germplasm analyzed in this study, geographical and environmental factors have a great effect in shaping the populations of *T. terrestris*.

### Implications for conservation and management

In the era of higher productivity and food security, many other plant species are being neglected, as the main land is used for cultivation of major crops and remaining lands are coming under various developmental processes. *T. terrestris* is one such plant which is frequently uprooted to replace major crops thus shrinking in minor patches. Sarwat et al. (2008) has already suggested for the conservation of diverse germplasm of this species. Furthermore, Mohammed et al. (2004) has included *T. terrestris* in the list of underutilized medicinal plants of Indian thar desert and suggested its commercial cultivation in this region. Although, its cultivation in dry areas can be taken as a conservation measure but duplication of same germplasm over the years can not ensure preservation of high genetic diversity in it. Further, duplicated germplasm thought to be less stable against any epidemic and therefore is not considered good in view of long-term usage and sustainable utilization. Although, high genetic diversity reported in *T. terrestris* here showed occurrence of valuable diverse germplasm in north India, continued negligence in some regions especially Punjab and Haryana can lead to loss of important germplasm lines. Thus, it needs attention towards conservation and introduction of diverse lines from fertile to dry regions where it is cultivated extensively to enhance genetic diversity and to ensure its conservation. To achieve this, it is suggested that introduction of various populations of *T. terrestris* from fertile parts of north India i.e. Punjab and Haryana should be done in north western dry parts of Rajasthan. This can result in conservation of diverse germplasm lines. Further, the demand of this herb in pharmaceutical industries will remain increasing in future, therefore, the abundantly existing resources of this species in north western dry regions needs to be utilized in a well planned way so that the industries relying on raw materials do not face any scarcity in coming years. Thus, by introducing diverse populations in north western parts will create ex situ conservatories for this species. In addition, utilization of existing resources of both fertile and dry regions in a planned manner can assure uninterrupted supply of this species to industries and its conservation for future.

## Conclusions

In conclusion, this was the first attempt of SSR development in *T. terrestris* and these markers appeared highly polymorphic and informative in characterized germplasm. Moreover, genetic diversity and population structure related studies are severely lacking in this species. Therefore, the novel information and SSR markers provided here can be useful in accelerating these types of studies in different germplasms of *T. terrestris.* We found that there is considerable genetic diversity prevailing in north Indian germplasm of *T. terrestris* which needs preservation can be exploited in a sustainable manner. The results of present study can be helpful in planning the utilization patterns and management of *T. terrestris* germplasm existing in north India. The novel SSR markers developed in this study can be useful in future genetic characterization related studies of the germplasm of this species.

## Electronic supplementary material

Below is the link to the electronic supplementary material.

Supplementary material 1 (DOCX 34 kb)

Supplementary material 2 (XLSX 12 kb)
