# Supplementary material for: Development of novel SSR markers for evaluation of genetic diversity and population structure in Tribulus terrestris L. (Zygophyllaceae)
Source: 3 Biotech. 2016 Jul 19;6(2):156. doi: 10.1007/s13205-016-0469-8 (PMC4951381; doi:10.1007/s13205-016-0469-8)
Supplement: Supplementary file 1 — Supplementary material 1 (DOCX 34 kb) [file 13205_2016_469_MOESM1_ESM.docx]

| **K** | **Reps** | **Mean LnP(K)** | **Stdev LnP(K)** | **Ln'(K)** | **\|Ln''(K)\|** | **Delta K** |
| --- | --- | --- | --- | --- | --- | --- |
| 1 | 3 | -1029.266667 | 0.450925 | — | — | — |
| **2** | **3** | **-823.033333** | **0.152753** | **206.233333** | **117.033333** | **766.163013** |
| 3 | 3 | -733.833333 | 0.650641 | 89.200000 | 44.766667 | 68.803974 |
| 4 | 3 | -689.400000 | 0.300000 | 44.433333 | 7.566667 | 25.222222 |
| 5 | 3 | -637.400000 | 1.212436 | 52.000000 | — | — |

**Supplementary table 1.** The Evanno table output from Structureharvester showing maximum value of delta k at K=2

**Supplementary Table 2**. The raw STRUCTURE output values of all the runs from 1-5 with 3 iterations.

| **File name** | **Run #** | **K** | **Est. Ln prob. of data** | **Mean value of Ln likelihood** | **Variance of Ln likelihood** |
| --- | --- | --- | --- | --- | --- |
| Resultstrbp_run_1_f | 1 | 1 | -1029.7 | -1019.9 | 19.6 |
| Resultstrbp_run_2_f | 2 | 1 | -1028.8 | -1019.6 | 18.5 |
| Resultstrbp_run_3_f | 3 | 1 | -1029.3 | -1019.8 | 18.9 |
| Resultstrbp_run_6_f | 6 | 2 | -823.2 | -795.9 | 54.5 |
| Resultstrbp_run_5_f | 5 | 2 | -822.9 | -796.1 | 53.6 |
| Resultstrbp_run_4_f | 4 | 2 | -823.0 | -796.0 | 53.9 |
| Resultstrbp_run_8_f | 8 | 3 | -733.2 | -701.3 | 63.8 |
| Resultstrbp_run_7_f | 7 | 3 | -734.5 | -701.4 | 66.3 |
| Resultstrbp_run_9_f | 9 | 3 | -733.8 | -701.4 | 64.9 |
| Resultstrbp_run_10_f | 10 | 4 | -689.1 | -646.6 | 84.9 |
| Resultstrbp_run_12_f | 12 | 4 | -689.7 | -641.9 | 95.5 |
| Resultstrbp_run_11_f | 11 | 4 | -689.4 | -646.4 | 85.9 |
| Resultstrbp_run_15_f | 15 | 5 | -636.7 | -586.4 | 100.7 |
| Resultstrbp_run_13_f | 13 | 5 | -638.8 | -586.2 | 105.1 |
| Resultstrbp_run_14_f | 14 | 5 | -636.7 | -586.2 | 101.0 |

**Supplementary Table 3.** Summary of AMOVA Table

| **Source** | **df** | **SS** | **MS** | **Est. Var.** | **%** |
| --- | --- | --- | --- | --- | --- |
| **Among Pops** | 2 | 38.718 | 19.359 | 1.678 | 24% |
| **Within Pops** | 23 | 124.667 | 5.420 | 5.420 | 76% |
| **Total** | 25 | 163.385 |  | 7.098 | 100% |


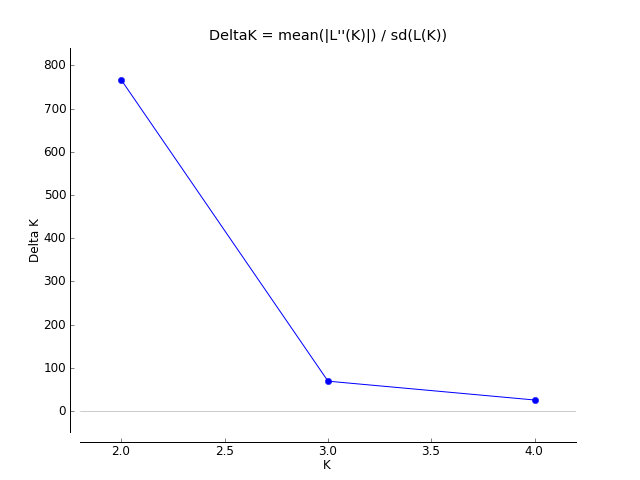


**Supplementary figure 1.** Estimation of K value by rate of change of delta k as per Evano et al.
